# Anaerobic capacity estimated by the sum of both oxygen equivalents from the glycolytic and phosphagen pathways is dependent on exercise mode: Running versus cycling

**DOI:** 10.1371/journal.pone.0203796

**Published:** 2018-09-13

**Authors:** Paulo Eduardo Redkva, Willian Eiji Miyagi, Fabio Milioni, Alessandro Moura Zagatto

**Affiliations:** 1 Post-Graduate Program in Movement Sciences, São Paulo State University (UNESP), Bauru, São Paulo, Brazil; 2 Laboratory of Physiology and Sports Performance (LAFIDE), Department of Physical Education, School of Sciences, São Paulo State University (UNESP), Bauru, São Paulo, Brazil; IRCCS E. Medea, ITALY

## Abstract

The purpose of this study was to verify whether the exercise modality (i.e., running and cycling) alters the magnitude of “anaerobic” capacity estimated by a single supramaximal effort (AC_[La]+EPOCfast_). Fourteen healthy men (age: 26±9 years) underwent a maximum incremental test and a supramaximal effort to exhaustion at 115% of the intensity associated with maximal oxygen uptake to determine the AC_[La]+EPOCfast_ (i.e., the sum of both oxygen equivalents from the glycolytic and phosphagen pathways), performed on both a treadmill and cycle ergometer. The maximal oxygen uptake during running was higher (p = 0.001; large effect size) vs. cycling (48.9±3.9mL·kg^-1^·min^-1^ vs. 44.8±5.5mL·kg^-1^·min^-1^ respectively). Contrarily, the oxygen equivalent from the glycolytic metabolism was not different between exercise modalities (p = 0.133; small effect size; running = 2.35±0.48 L and cycling = 2.18±0.58 L). Furthermore, the “anaerobic” capacity was *likely meaning fully* (3.65±0.70 L) and *very likely meaningfully* (949.1±5.7 mL·kg^-1^) greater in running than cycling (3.81±0.71 L and 52.0±8.1 mL·kg^-1^). Additionally, the contribution of the phosphagen metabolism was higher (p = 0.001; large effect size) for running compared to cycling (1.6±0.3 L vs.1.3±0.3 L respectively). Therefore, the “anaerobic” capacity estimated by the sum of both oxygen equivalents from the glycolytic and phosphagen pathways during a supramaximal effort is influenced by exercise modality and is able to identify the difference in phosphagen metabolic contribution, based on the methodological conditions of this study.

## Introduction

Physiological responses, such as blood lactate concentration ([La]), oxygen uptake ($\dot{\text{V}}{\text{O}}_{2}$), $\dot{\text{V}}{\text{O}}_{2}$ slow component, and kinetics *Off* $\dot{\text{V}}{\text{O}}_{2}$ responses [1] are significantly altered by different exercise modalities (e.g., treadmill, cycle ergometer, rowing, and swimming) [2,3], due to variables which are inherent to each mode (i.e., active muscle mass, body position, motor pattern, and others). In addition, the magnitute of the effect of the exercise modality on physiological responses seems to be more evidencied during maximal intensities [3], thus, some metabolic parameters widely used to assess physical fitness (i.e., maximal oxygen uptake and maximal accumulated oxygen deficit) are also modified [4].

Regarding “anaerobic” assessment (i.e., non-mitochondrial metabolic pathways), the maximal accumulated oxygen deficit (MAOD) is considered the most accepted method to assess “anaerobic” capacity [2,5] and seems to be affected by the exercise modality [4,6,7]. Hill and Vingren [7] described that in moderately active women and men, the MAOD estimated in running is greater compared with cycling possibly due to the greater muscle mass that is active during running. In addition, Billat and co-workers [8] reported that the $\dot{\text{V}}{\text{O}}_{2}$ slow component is higher during cycling compared with running, which alters the linear intensity-$\dot{\text{V}}{\text{O}}_{2}$ relationship and consequently, MAOD determination. Thus, it is possible to infer the effect of muscle mass on MAOD assessment.

As a way of optimizing the “anaerobic” capacity estimation, Bertuzzi et al. [9] proposed an alternative method for assessing “anaerobic” capacity in a single supramaximal effort (AC_[La]+EPOCfast_) based on the sum of oxygen equivalents from the phosphagen (E_PCr_) and glycolytic (E_[La]_) energy pathways, describing that AC_[La]+EPOCfast_ is similar and correlated with MAOD. The AC_[La]+EPOCfast_ procedure proposed by Bertuzzi et al. [9] estimates the “anaerobic” capacity based exclusively on the fast component of excess post-exercise oxygen consumption (EPOC_fast_), enabling estimation of the E_PCr_, and on *delta* blood lactate concentration (i.e., peak lactate value minus baseline lactate value; Δ[La]), enabling estimation of the E_[La]_, using methods proposed by Margaria et al. [10] and di Prampero and Ferretti [11], respectively.

In addition to the aforementioned study of Bertuzzi et al. [9], Zagatto et al. [12] and Miyagi et al. [13] reinforced the validity of AC_[La]+EPOCfast_ for running and cycling respectively, showing that AC_[La]+EPOCfast_ was also similar to MAOD and adding that exercise intensity at 115% of the intensity associated with maximal oxygen uptake ($\text{i}\dot{\text{V}}{\text{O}}_{\text{2max}}$) corresponded to the greatest intensity to determine the AC_[La]+EPOCfast_.

However, as the AC_[La]+EPOCfast_ procedure is a recent method, further studies investigating certain factors that could affect the blood lactate response or excess post-exercise oxygen consumption are necessary, such as the active muscle mass during effort and consequently the effect of ergometers specificities. According to reports in the literature, running involves a greater amount of active muscles [14] and a lower magnitude of the $\dot{\text{V}}{\text{O}}_{2}$ slow component [2] compared to cycling [1,14], leading to an increased area from the EPOC_fast_ [1]. It is also known that active muscle mass affects blood lactate concentration [7], and would consequently affect the glycolytic responses used to assess AC_[La]+EPOCfast_. In addition, as the “anaerobic” capacity is defined as the maximal amount of adenosine triphosphate resynthesized via non-mitochondrial pathways during a specific mode of short-duration maximal exercise [15], the “anaerobic” capacity must be measured in specific exercise testing according to training and sport modality. Therefore, it is hypothesized that different values of AC_[La]+EPOCfast_ will be found when estimated in running and cycling. In addition, due the differences in muscles mass involved [2] and motion patterns specificities [4] of each modality, it is also expected different contributions of the energy systems between running and cycling, which are ergometers widely used to evaluate the physical fitness and like training mode. Therefore, the purpose of this study was to verify whether the exercise modality, i.e., running vs. cycling, affects the magnitude of AC_[La]+EPOCfast_.

## Materials and methods

### Subjects

Fourteen healthy male, (mean±SD: age 26±9 years, height 174.1± 4.9 cm, body mass 72.9±10.8 kg, body fat percentage 16.5±4.1%), participated voluntarily in this study. The individuals were involved in recreational physical exercise, but none were classified as trained and none were especially experienced in cycling or running. All participants were informed of the experimental risks and signed an informed consent form prior to the investigation in accordance with the Declaration of Helsinki. The experimental procedures, as well as the informed consent, were approved by the Research Ethics Committee of the Sao Paulo State University, Brazil (Protocol number 645.784/2014).

### Experimental design

**Fig 1:**
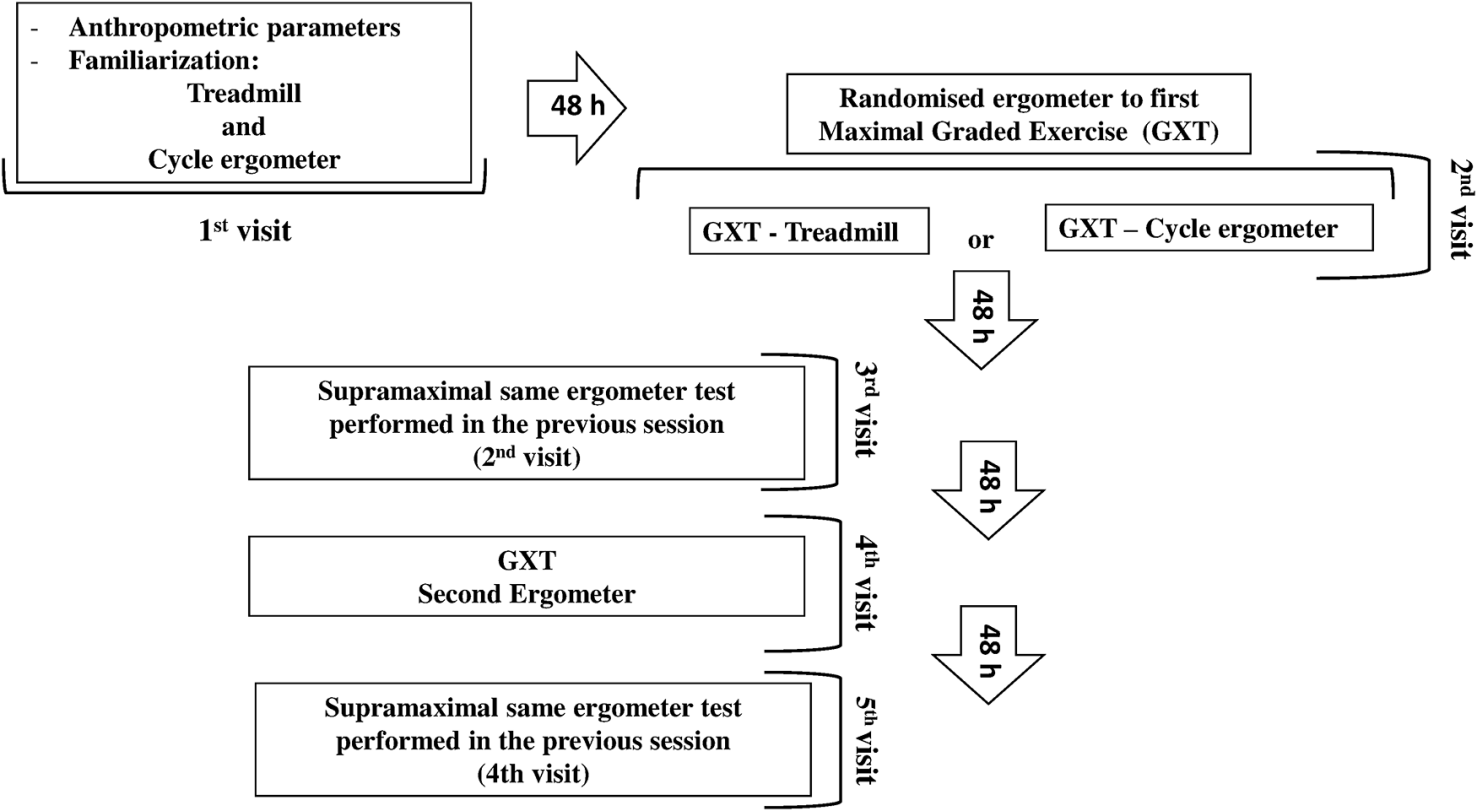
The study design required participants to attend the laboratory on five separate occasions. The initial visit consisted of anthropometric measurements and familiarization with the ergometers; the second and fourth visits were carried out the randomized graded exercise test on ergometers and the remaining two visits were carried out the supramaximal tests.

The participants performed five experimental trials that were separated by an interval of at least 48-h for recovery (Fig 1). The volunteers were required to refrain from exhaustive exercise, and alcohol and caffeine ingestion for 48-h prior to data collection. To eliminate any influence of circadian rhythm, each subject completed all trials at the same time period of the day in controlled environmental conditions regarding temperature (22.9 ± 1.3°C) and relative humidity (43.8 ± 6.3%). Participants were instructed to maintain the same diet throughout the study.

Each participant performed the procedures on the electromagnetic braking cycle ergometer (Excalibur Sport, Lode, Groningen, Netherlands) and motorized treadmill (ATL, Inbramed, Inbrasport, Porto Alegre, RS, Brazil). During the first session, the body composition was measured by Dual Energy X-ray Absorptiometry (Discovery, Hologic, USA), followed by familiarization on the cycle ergometer (10 min at 100 W) and motorized treadmill (10 min at 8 km·h^-1^). The initial sequence of ergometers was randomized (motorized treadmill or cycle ergometer) and assessments for each ergometer were conducted in two consecutive sessions for the same ergometer.

Initially, a graded exercise test was performed to determine the maximal oxygen uptake ($\dot{\text{V}}{\text{O}}_{\text{2max}}$) and the minimal intensity at which $\dot{\text{V}}{\text{O}}_{\text{max}}$ was reached ($\text{i}\dot{\text{V}}{\text{O}}_{\text{2max}}$). Next, a supramaximal test was performed until voluntary exhaustion at 115% of $\text{i}\dot{\text{V}}{\text{O}}_{\text{2max}}$ (each ergometer) to determine AC_[La]+EPOCfast_ [12,13]. For each test, the participants were verbally encouraged to perform maximal effort. For the treadmill running efforts, participants wore a chest harness with the rope attached to the ceiling to ensure maximal effort without the risk of falling.

Prior to each testing effort, the warm-ups were standardized at 100 W on the cycle ergometer and 8 km·h^-1^ on the treadmill, lasting five minutes. The tests started five minutes after the end of the warm-up.

### Procedures

#### Measurement of physiological and metabolic parameters

During all tests, the respiratory responses were measured breath-by-breath using a stationary gas analyzer (Quark PFT, COSMED, Rome, Italy). The gas analyzer was calibrated before each test using an ambient air sample and a high-precision gas mixture (3.98% CO_2_, 16.02% O_2_ and balanced N_2_; White Martins Gases Industrials Ltda, Osasco, SP, Brazil), whereas the turbine was calibrated before each test and verified after each test using a 3-L calibration syringe (Hans-Rudolf, Kansas City, MO, USA) in accordance with the manufacturer’s instructions. In addition, in supramaximal efforts, the $\dot{\text{V}}{\text{O}}_{2}$ was measured for 10 min at rest (i.e., before warm-up) for the baseline assessment and for 7 minutes after the end of the test to assess the EPOC_fast_. For analysis of respiratory variables, data were smoothed every 5-s and interpolated every 1 second. Heart rate (HR) was measured by means of a transmitter belt coupled to the gas analyzer (Wireless HR Monitor, COSMED, Rome, Italy).

Blood samples were collected 3, 5, and 7 minutes after each effort and the highest [La] measured was assumed as the peak value for each test. In the supramaximal effort, blood samples were also collected at rest before any physical effort (i.e., after 10 minutes sitting) to measure the baseline lactate concentration. Blood samples (25μL) were collected from the ear lobe using heparinized capillary tubes and transferred to *Eppendorf* tubes containing 50μL of 1% sodium fluoride for subsequent electrochemical analysis of lactate (YSI 2300 STAT, Yellow Spring Instruments, Yellow Spring, Ohio, USA).

#### Maximal graded exercise tests

The graded exercise test (GXT) on the treadmill began at 8 km·h^−1^ with staged increments of 1.5 km·h^−1^ every 2 min until exhaustion (gradient set at 1%) [12,16]. The GXT in cycling started with an intensity corresponding to 100 W, with increments of 25 W each 2 min stage of the exercise until voluntary exhaustion or until the inability of the individual to maintain a cadence of 70–75 revolutions per minute (rpm). The graded exercise tests were designed to last between 8–12 min.

The Borg scale (6–20) [17] was used to assess the rating of perceived exertion (RPE) at the end of each stage of the GXT tests. The highest average of the $\dot{\text{V}}{\text{O}}_{2}$ (i.e., average of the $\dot{\text{V}}{\text{O}}_{2}$ during the final 30-s of each stage) attained during the test was considered as $\dot{\text{V}}{\text{O}}_{\text{2max}}$ [18]considering the verification of a plateau in $\dot{\text{V}}{\text{O}}_{2}$ (variation in $\dot{\text{V}}{\text{O}}_{2}$ < 2.1 mL·kg^−1^·min^−1^ between the final and penultimate stage of exercise). As secondary criteria to consider $\dot{\text{V}}{\text{O}}_{\text{2max}}$, at least two of the following criteria were required to be observed: maximal HR (HR_max_) ≥ 90% of predicted HR_max_; respiratory exchange ratio (RER) ≥ 1.10; and peak lactate ≥ 8.0 mmol·L^−1^ [18]. If $\dot{\text{V}}{\text{O}}_{2}$ plateau or at least two criteria were not observed, a new test was applied. The exercise velocity (for treadmill) or power output (for cycler ergometer) at which the subject reached $\dot{\text{V}}{\text{O}}_{\text{2max}}$ was considered as $\text{i}\dot{\text{V}}{\text{O}}_{\text{2max}}$ for each ergometer. If the final stage had not been completed, the $\text{i}\dot{\text{V}}{\text{O}}_{\text{2max}}$ was calculated using the equation proposed by Kuipers et al. [19].

#### Estimation of “Anaerobic” capacity through AC_[La+EPOCfast]_ method

The AC_[La+EPOCfast]_ was determined as suggested by Bertuzzi et al. [9] and considering the exercise intensity reported by Zagatto et al. [12] and Myiagi et al. [13].

Supramaximal efforts were performed at an intensity corresponding to 115% of $\text{i}\dot{\text{V}}{\text{O}}_{\text{2max}}$ determined for each ergometer [12,13]. The highest average of the $\dot{\text{V}}{\text{O}}_{2}$ during the final 20-s of supramaximal effort was considered as exhaustion $\dot{\text{V}}{\text{O}}_{2}\;(\dot{\text{V}}{\text{O}}_{\text{2EX}}$). The AC_[La+EPOCfast]_ was assumed as the sum of the E_PCr_ and E_[La]_ [9,12,13]. In addition, the time-to-exhaustion was measured.

The EPOC_fast_ was used to estimate the contribution of the E_PCr_, which was calculated using a bi-exponential fit [9,12,13]in OriginPro 9.0 software (OriginLab Corp., Microcal, Mass., USA)

$${\dot{\text{V}}\text{O}}_{2(\text{t})}=\dot{\text{V}}{\text{O}}_{2\text{baseline}}\text{+A1[}{\text{e}}^{\text{-(t-}\delta \text{)/}\tau \text{1}}]+\text{A}2[{\text{e}}^{\text{-(t-}\delta \text{)/}\tau 2}\text{]}$$

where $\dot{\text{V}}{\text{O}}_{2(\text{t})}$ is the rate of oxygen uptake at time (t); $\dot{\text{V}}{\text{O}}_{2\text{baseline}}$ is the rate of oxygen uptake at baseline; A is the amplitude; δ is the time delay; and τ is the time constant– 1 and 2 represent the fast and slow components, respectively–and EPOC_fast_ was calculated by the product of A1 and τ1.

The contribution of the E_[La]_ was estimated by the difference between the quantities of blood lactate concentration at peak and rest (i.e., Δ[La]), considering each 1 mmol·L^−1^ of accumulated lactate as equivalent to 3 mL O_2_·kg^−1^ [11].

### Statistical analyses

Data are presented as means ± SD and confidence interval of 95% (CI95%). Initially, the Shapiro-Wilk test was used to verify the normality of the data. Next, the *t*-test for dependent samples was used to compare the variables obtained on the motorized treadmill and cycle ergometer. In addition, the effect size (ES) was calculated considering the threshold values for Cohen’s *d* statistical power as ≥0.2 (small), ≥0.5 (moderate), and ≥0.8 (large). The Pearson’s correlation test was used to verify the association between the variables. The coefficient of correlation was classified as very weak to negligible (0 <0.2), weak (0.2 <0.4), moderate (0.4 <0.7), strong (0.7 <0.9), and very strong (0.9 <1.0). Statistical significance was accepted when p<0.05.

As alternative analysis, magnitude-based inference analysis was also used. The raw outcomes were log-transformed before analysis to reduce non-uniformity of error[20]. Magnitude-based inference was used to determine the practical significance and smallest worthwhile changes (non-clinical inference) in the comparison of scores between the cycle ergometer and treadmill, using the method described by [21]. A Cohen’s unit of 0.2 was used to determine the smallest worthwhile value of change. Using a Microsoft Excel^®^ spreadsheet designed for sports science research [22], mean effects and 90% confidence limits were estimated to establish the percentage likelihood of each experimental condition having a negative/trivial/positive effect. Thus, the changes in benefit were qualitatively evaluated as follows: 0.5%–5% = *very unlikely*; 5%–25% = *unlikely*; 25%–75% = *possibly*; 75%–95% = *likely*; 95%–99.5% = *very likely*; and >99.5% = *most likely* [20]. When the positive and negative values were both >5%, the inference was classified as *unclear*.

## Results

All subjects reached the exhaustion criteria in the GXT and did not need to repeat the test. The time-to-exhaustion in the GXT was 12.8±3.1 min (CI95% = 10.9 to 14.5 min) on the cycle ergometer and 9.7±1.6 min (CI95% = 8.7 to 10.6 min) on the treadmill. The $\dot{\text{V}}{\text{O}}_{\text{2max}}$ determined in the cycling was lower compared with the running (p = 0.018), whereas the values of [La] peak obtained in the GXT were higher in the cycling (p = 0.012). Physiological parameters measured during the GXT are presented in Table 1.

**Table 1:** Physiological variables determined in the graded exercise test on the cycle ergometer and motorized treadmill (n = 14).

Variable	Cycling	Running	p-values
$\dot{\text{V}}{\text{O}}_{\text{2max}}$ **(mL**·**kg**^**-1**^·**min**^**-1**^**)**	44.7 ± 5.7 (41.5 to 48.0)	49.2 ± 3.8** (47.0 to 51.4)	0.018
$i\dot{\text{V}}{\text{O}}_{\text{2max}}$ **(W)**	233.3 ± 38.4 (211.2 to 255.55)	-	-
$i\dot{\text{V}}{\text{O}}_{\text{2max}}$ **(km**·**h**^**-1**^**)**	-	13.7 ± 1.3 (13.0 to 14.4)	-
**RER peak**	1.19 ± 0.07 (1.14 to 1.23)	1.16 ± 0.05 (1.13 to 1.20)	0.188
**HR**_**max**_ **(bpm)**	184.3 ± 6.4 (180.6 to 188.0)	188.1 ± 8.7** (183.1 to 193.2)	0.008
**[La] peak (mmol**·**L**^**-1**^**)**	10.0 ± 1.5 (9.1 to10.8)	8.8 ± 1.7** (7.8 to 9.9)	0.012

Values in means ± SD (CI95%). $\dot{\text{V}}{\text{O}}_{\text{2max}}$ = Maximal rate of oxygen uptake. $i\dot{\text{V}}{\text{O}}_{\text{2max}}$ = Lowest intensity corresponding to the $\dot{\text{V}}{\text{O}}_{\text{2max}}$. RER peak = Peak respiratory exchange ratio. HR_max_ = Maximum heart rate. [La] peak = Lactate peak concentration.

*p< 0.05 in relation to the cycle ergometer.

**p< 0.01 in relation to the cycle ergometer.

**Fig 2:**
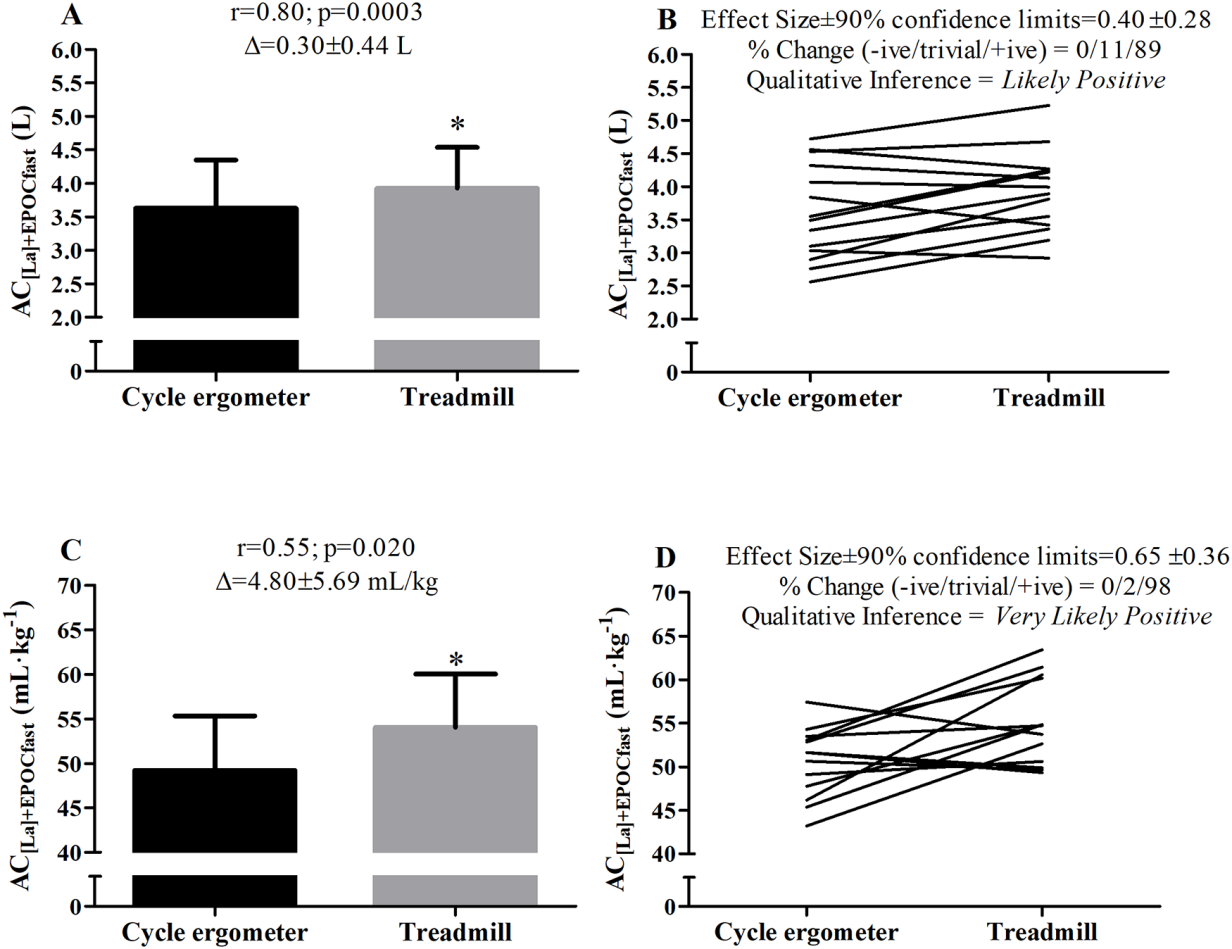
Comparison of the “anaerobic” capacity by blood lactate and EPOC_fast_ (AC_[La]+EPOCfast_) values determined during cycling and running. Left panels (A and C) correspond to mean and standard deviation values and Right panels (B and D) correspond to individual values. *p<0.05 compared to the cycling.

The results of the supramaximal effort at 115% of $\text{i}\dot{\text{V}}{\text{O}}_{\text{2max}}$ determined in the cycling and running are presented in Table 2 and in Fig 2. The $\dot{\text{V}}{\text{O}}_{\text{2EX}}$ in cycling [43.3 ± 4.0 mL·kg^-1^·min^-1^ (CI 95% = 41.3 to 45.7 mL·kg^-1^·min^-1^)] was higher (p = 0.006) than running [48.0 ± 4.2 mL·kg^-1^·min^-1^ (CI 95% = 45.6 to 50.6 mL·kg^-1^·min^-1^)]. No significant differences were found for time-to-exhaustion for cycling [175.9 ± 22.0 s (CI 95% = 163.2 to 188.7 s)] and running [155.4 ± 43.2 s (CI 95% = 130.5 to 180.4 s)] (p = 0.114). The phosphagen pathway outcomes (A1, τ-1, and E_PCr_) were higher in the running compared with cycling(p≤0.04), except for blood lactate responses, oxygen equivalent estimated from the glycolytic pathway, and RPE. These statistical findings were also reported by magnitude-based inference analysis, describing higher meaningful values when measured in running, except for the E_[La]_ expressed in absolute values (Liters of oxygen) which demonstrated a *possibly negative* inference (i.e., higher value in cycling), although the possibility of change was only 27%. In addition, significant correlations were found between parameters measured during cycling and running for blood lactate, E_[La]_, A_1_, E_PCr_, and RPE.

**Table 2:** Comparison and relationship between the variables obtained in the supramaximal intensity efforts at 115% of $\text{i}\dot{\text{V}}{\text{O}}_{\text{2max}}$ on the cycle ergometer and motorized treadmill.

Variables	Cycling	Running	p-value	Δ%	Effect size (Cohen’s*d±*90%CL)	%Chances (Negative/ Trivial/ Positive)	Qualitative inference	Correlation Coefficient (95%CI)
**[La] peak (mmol**·**L**^**-1**^**)**	11.6 ± 1.6 (10.7 to 12.5)	11.5 ± 2.1 (10.28 to 12.7)	0.811	-0.09±1.36	-0.09±0.33	28/65/7	*Unclear*	0.76** (0.20 to 0.86)
**[La] rest (mmol**·**L**^**-1**^**)**	1.1 ± 0.4 (0.8 to 1.3)	1.0 ± 0.3 (0.8 to 1.2)	0.676	-0.04±0.36	-0.04±0.38	23/62/15	*Unclear*	0.59* (0.18 to 0.85)
**Δ[La]** **(mmol**·**L**^**-1**^**)**	10.5 ± 1.7 (9.6 to 11.5)	10.5 ± 2.1 (9.2 to 11.7)	0.884	-0.05±1.21	-0.06±0.27	5/76/18	*Likely Trivial*	0.82** (0.20 to 0.86)
**E**_**[La]**_ **(L)**	2.33 ± 0.49 (2.04 to 2.62)	2.27 ± 0.51 (1.97 to 2.57)	0.501	-0.06±0.31	-0.11±0.26	27/70/3	*Possibly negative*	0.816** (0.28 to 0.88)
**E**_**[La]**_ **(mL**·**kg**^**-1**^**)**	31.6 ± 5.0 (29.0 to 34.5)	31.4 ± 6.3 (27.8 to 35.1)	0.884	-0.14±3.62	-0.06±0.27	18/76/5	*Likely Trivial*	0.82** (0.21 to 0.86)
**A**_**1**_ **(mL**·**kg**^**-1**^·**min**^**-1**^**)**	19.3 ± 2.2 (18.0 to 20.5)	20.4 ± 1.7** (19.4 to 21.3)	0.045	+1.06±1.78	0.44±0.35	0/12/88	*Likely Positive*	0.605* (0.23 to 0.87)
**τ-1 (min)**	0.91 ± 0.10 (0.85 to 0.97)	1.12 ± 0.13** (1.04 to 1.19)	0.001	+0.21±0.18	1.76±0.70	0/0/100	*Most likely Positive*	-0.13 (-0.61 to 0.36)
**E**_**PCr**_ **(L)**	1.29 ± 0.34 (1.1 to 1.5)	1.64 ± 0.27** (1.5 to 1.8)	0.0001	+0.35±0.23	0.93±0.32	0/0/100	*Most likely Positive*	0.74* (0.39 to 0.90)
**E**_**PCr**_ **(mL**·**kg**^**-1**^**)**	17.7 ± 3.2 (15.8 to 19.5)	22.6 ± 2.2** (21.3 to 23.8)	0.0001	+4.94±3.37	1.28±0.44	0/0/100	*Most likely Positive*	0.27 (-0.26 to 0.67)
**RPE** **(arbitrary unit)**	18 ± 2 (16.1 to 18.7)	17 ± 2 (16.6 to 18.7)	0.819	+0.08±1.19	0.05±0.25	5/79/15	*Likely Trivial*	0.83* (0.47 to 0.93)

Values in means ± SD (CI95%). [La] peak = Lactate peak concentration. [La] rest = Lactate rest concentration E_[La]_ = contribution of the glycolytic metabolism. Δ[La] = difference between the lactate peak and rest. E_PCr_ = contribution of the phosphagen metabolism. A_1_ = amplitude 1 the bi-exponential adjustment. τ-1 = constant time1 the bi-exponential adjustment. RPE = rate of perceived exertion. ES = Effect Size. Δ% = percentage alteration. The quantitative chances were assessed qualitatively as follow: 0.5%–5% = very unlikely; 5%–25% = unlikely; 25%–75% = possibly; 75%–95% = likely; 95%–99.5% = very likely; and >99.5% = most likely. If the probabilities of the effect being substantially positive and negative were both > 5%, the effect was reported as unclear.

* = p< 0.05 in relation to the cycle ergometer.

** = p< 0.01 in relation to the cycle ergometer.

Concerning the “anaerobic” capacity magnitude estimated by AC_[La+EPOCfast]_, higher values were also found in running compared with cycling (Fig 2), these findings being reinforced by the magnitude-based inference analysis that reported a *likely positive* effect of AC_[La+EPOCfast]_ determined in running (89% chance of positive effect for AC_[La+EPOCfast]_ expressed in absolute values and 98% when the value was expressed relative to body mass). In addition, a significant correlation was found (p = 0.0003 for AC_[La+EPOCfast]_ expressed in absolute values and p = 0.020 when the value was expressed relative to body mass) between AC_[La+EPOCfast]_ determined in the different exercise modalities. These results, as well as the individual AC_[La+EPOCfast]_ data are showed in Fig 2. All raw data are presented at S1 Data spreadsheet.

## Discussion

The present study aimed to verify whether the exercise modality affects the AC_[La+EPOCfast]_. The main findings were that in moderately active subjects, running on a treadmill allowed greater energetic contribution from the phosphagen metabolism pathway and higher “anaerobic” capacity, evidenced by AC_[La+EPOCfast]_. Conversely, the glycolytic metabolism pathway was not different in cycling and running. Based on these results, the initial hypothesis of the study (i.e., exercise modality would alter the AC_[La+EPOCfast]_ as well as the energy systems contributions) was confirmed.

There are some studies in the literature reporting the effects of exercise modality on conventional MAOD [4,6,7]. The higher MAOD value in running can be attributed to the greater energy demand during supramaximal effort in this type of exercise [23], largely due to the greater muscle mass activated [7]. In fact, higher MAOD values are expected because the “anaerobic” capacity is related to the amount of muscle mass that is active during effort [4].

The “anaerobic” capacity estimated by AC_[La+EPOCfast]_ is determined using a different method. While the conventional MAOD needs to apply several submaximal trials to fit a linear $\dot{\text{V}}{\text{O}}_{2}$-intensity relationship, followed by a supramaximal effort to determine the accumulated oxygen deficit, in the AC_[La+EPOCfast]_ the “anaerobic” capacity is estimated by the sum of the oxygen equivalents from the phosphagen and glycolytic pathways[9,12,13]. In addition to feasibility considering time-required efficiency, this method is also able to distinguish between contributions from the phosphagen and glycolytic metabolism pathways in its calculation. The “anaerobic” capacity estimated by AC_[La+EPOCfast]_ was ~10% higher in running, while the E_PCr_ was ~32% higher in running.

Some authors have described that mode of exercise can overestimate (i.e., uphill exercise) or underestimate (i.e., cycling) the magnitude of MAOD [4]. In addition, in the same exercise modality, such as running, the exercise performed uphill can reflect in greater MAOD values compared with horizontal running [2], mainly due to the different set of muscles used during horizontal running [24]. However, it is important to consider the definition of “anaerobic” capacity described by Green [15], assumed as *“the maximal amount of ATP resynthesized via the anaerobic metabolism during a specific type of short-duration*, *maximal exercise*”. Therefore, it is plausible to assume that the “anaerobic” capacity estimated by AC_[La+EPOCfast]_ is ergometer-dependent and seems to be specific to exercise modality performed, instead of assuming that it is overestimated (i.e., running) or underestimated (i.e., cycling).

The procedure used to estimate each energetic contribution was responsive to identify statistical differences in E_PCr_ between ergometers (Table 2). During the initial phase of EPOC_fast_, restoration of phosphocreatine stores is through metabolic processes that rely on $\dot{\text{V}}{\text{O}}_{2}$ [11]. As the E_PCr_ is estimated based on $\dot{\text{V}}{\text{O}}_{2}$ amplitude and constant time for oxygen deficit (τ-1), a higher $\dot{\text{V}}{\text{O}}_{2}$ response during effort in running results in a higher $\dot{\text{V}}{\text{O}}_{2}$ amplitude and τ-1, with consequently greater E_PCr_. Some studies have used 7 min [13,25], ~10 min or until the values return to the rest values [9]. However, these studies have used mathematical adjustments similar to the present study and similar τ-1 values as well. In the study of Miyagi et al. [13], the τ1 values were 1.00±0.21 min and 1.09±0.20 min in cycling test-retest whereas Zagatto et al. [25] the values of τ-1 were 1.00±0.13 min in treadmill. Therefore, although the recovery time after the test may influence the results, in the present study the results were similar to those in the literature and did not appear to have been harmed.

As exercise during cycling and running involves different active muscle mass [6], this difference can promote a relationship between the $\dot{\text{V}}{\text{O}}_{2}$ response and actions of the involved muscles, with consequences in the different responses attributed to EPOC_fast_ [1]. Carter et al. [26] showed that A_1_ was higher in running compared with cycling at different intensities, supporting the findings of the present study (Table 2). Greater A_1_ in running revealed that $\dot{\text{V}}{\text{O}}_{2}$ in the relationship at 115% of $\text{i}\dot{\text{V}}{\text{O}}_{\text{2max}}$ was higher when compared to cycling (evident in the ratio of peak $\dot{\text{V}}{\text{O}}_{\text{2EX}}$ shown in Table 2), which implies different absolute values of $\dot{\text{V}}{\text{O}}_{2}$ between different exercise modalities. In addition, it is possible to speculate that, the higher active muscle mass during running likely improves the use of phosphocreatine stored in muscle, resulting in greater overall E_PCr_ for running compared to cycling.

In contrast, the E_[La]_ measurement was not statistically different between modalities, as well as the peak and Δ[La] (Table 2). The absence of differences for E_[La]_ between modalities might be, again, result of the muscle mass involved in cycling and running. While it was expected higher [La] for running due the greater muscle mass involved [7], that also may play an important role in lactate clearance capacity during the supramaximal effort (i.e., greater muscle mass involved) [27]. It has been shown that modes of exercise involving greater muscle mass during moderate exercise may present lower values of lactate for the same relative intensity due to greater lactate removal by the muscles involved in the task [27]. In addition, despite non-significant (p = 0.114), the time to exhaustion presented a clear trend to be greater in cycling than in running at supramaximal effort (175.9 ± 22.0 s vs 155.4 ± 43.2 s), which could have contributed to the similar values of peak [La] (since cycling involves less muscle mass than running).

One possible limitation of the present study is related to the study subjects; all healthy individuals who reported practicing recreational soccer, running, and cycling. Therefore, future investigations using the AC_[La+EPOCfast]_ for maximum running and cycling efforts in specific populations of athletes, i.e., triathletes, cyclists, and runners that present higher performance on their specific training ergometers, would be of great value to sport science. It is worth noting that the protocols were only performed after previous familiarization with both ergometers, aiming to minimize the influences of the exercise modality. Another limitation might be associated with the equivalent of O_2_ in relation to [La] used in this research. This relationship cannot represent the exact stoichiometric relationship between the formation of lactate and ATP resynthesis [9].

## Conclusion

We conclude that the exercise modality (running or cycling) affects the magnitude of “anaerobic” capacity determined thought AC_[La+EPOCfast]_. Additionally, in moderately active subjects, running on a treadmill allowed greater energetic contribution from the phosphagen metabolism pathway. Conversely, the glycolytic metabolism pathway was not different in cycling and running. This result, besides representing the need to be evaluated each modality in the respective ergometer avoiding transferences, it also enables athletes and coach to plan the training schedule according to the specific adaptation.

## Supporting information

S1 Data Raw data. (XLSX)
