# Correction: Anaerobic capacity estimated by the sum of both oxygen equivalents from the glycolytic and phosphagen pathways is dependent on exercise mode: Running versus cycling

**DOI:** 10.1371/journal.pone.0209884

**Published:** 2018-12-20

**Authors:** Paulo Eduardo Redkva, Willian Eiji Miyagi, Fabio Milioni, Alessandro Moura Zagatto

In the abstract, the values of data are incorrect. The full corrected abstract should read: The purpose of this study was to verify whether the exercise modality (i.e., running and cycling) alters the magnitude of “anaerobic” capacity estimated by a single supramaximal effort (AC_[La]+EPOCfast_). Fourteen healthy men (age: 26±9 years) underwent a maximum incremental test and a supramaximal effort to exhaustion at 115% of the intensity associated with maximal oxygen uptake to determine the AC_[La]+EPOCfast_ (i.e., the sum of both oxygen equivalents from the glycolytic and phosphagen pathways), performed on both a treadmill and cycle ergometer. The maximal oxygen uptake during running was higher (p = 0.001; large effect size) vs. cycling (49.2±3.8 mL·kg^-1^·min^-1^ vs. 44.7±5.7 mL·kg^-1^·min^-1^, respectively). Contrarily, the oxygen equivalent from the glycolytic metabolism was not different between exercise modalities (p = 0.133; small effect size; running = 2.27±0.51 L and cycling = 2.33±0.49 L). Furthermore, the “anaerobic” capacity was *likely meaningfully* (3.9±0.6 L and 54.1±6.0 mL·kg^-1^) and *very likely meaningfully* greater in running than cycling (3.6±0.7 L and 49.2±6.1 mL·kg). Additionally, the contribution of the phosphagen metabolism was higher (p = 0.001; large effect size) for running compared to cycling (1.6±0.3 L vs.1.3±0.3 L respectively). Therefore, the “anaerobic” capacity estimated by the sum of both oxygen equivalents from the glycolytic and phosphagen pathways during a supramaximal effort is influenced by exercise modality and is able to identify the difference in phosphagen metabolic contribution, based on the methodological conditions of this study.
